# A new feather mite species of the genus *Proterothrix* Gaud, 1968 (Acarina, Proctophyllodidae) from the Large Niltava, *Niltava grandis* (Passeriformes, Muscicapidae) – an integrative description

**DOI:** 10.3897/zookeys.661.11793

**Published:** 2017-03-14

**Authors:** Ioana Cristina Constantinescu, Oana Paula Popa, Luis Ovidiu Popa, Ioana Cobzaru, D. Khlur B. Mukhim, Costică Adam

**Affiliations:** 1 “Grigore Antipa” National Museum of Natural History, Sos. Kiseleff no.1, 011341 Bucharest 1, Romania; 2 Ecology, Taxonomy and Nature Conservation Department, Institute of Biology, Romanian Academy, Splaiul Independenței no. 296, 060031 Bucharest, Romania; 3 Department of Zoology, Lady Keane College, 793001 Shillong, Meghalaya, India

**Keywords:** Taxonomy, feather mite, new species, Pterodectinae, *Proterothrix*

## Abstract

A new species of the feather mite genus *Proterothrix* (Proctophyllodidae: Pterodectinae) is described from the Large Niltava *Niltava grandis* (Blyth) (Passeriformes, Muscicapidae) in northeast India (Meghalaya, Jaintia Hills, Shnongrim village). *Proterothrix chachulae* Constantinescu, **sp. n.** differs from all known species of the genus by having in males the aedeagus with bilobate tip. The morphological description is supplemented with molecular characterisation of a fragment f near the 5` terminus of the mitochondrial COI gene.

## Introduction

The bird genus *Niltava* Hodgson belongs to the family of Old World Flycatchers (Passeriformes: Muscicapidae), currently includes six valid species and is distributed in Indo-Malayan biogeographic region (Clements et al. 2016). Feather mites were previously recorded only in two of these species: *Analges* sp., *Anisodiscus* sp., *Mesalgoides* sp., *Proctophyllodes cotyledon* Trouessart, 1899, *Bicentralges distinctus* Orwig, 1968, *Proterothrix* sp., and *Trouessartia* sp. ex *Niltava grandis* (Blyth); *Analges* sp., *Proctophyllodes elegans* Atyeo and Braasch, 1966, *Proterothrix* sp., *Therisalges* sp., *Trouessartia* sp., and *Xolalges* sp. ex *Niltava sundara* Hodgson (Atyeo 1973; Atyeo and Braasch 1968; Orwig 1968). An unidentified *Proterothrix* species, reported by Atyeo (1973) from *Niltava grandis* in Asia is a potentially a new species, but has never been described. In this paper we describe a new species of *Proterothrix* collected from *Niltava grandis grandis* in Meghalaya (Northeast India).

The genus *Proterothrix* Gaud, 1968 (Proctophyllodidae: Pterodectinae) includes 28 species known exclusively from the Old World (Ethiopian, Oriental and Australasian regions) (Hernandes and Valim 2014). In the Oriental region, the genus appears to be one of the most widespread proctophyllodid genera, with 13 described species (from Malaysia, Taiwan, China, Vietnam and India) and 56 potentially new species recorded by Atyeo (1973), but never described elsewhere.

Species of the genus *Proterothrix* have been identified to date on birds from the orders Coraciiformes (Alcedinidae) and Passeriformes (Dicruridae, Eurylaimidae, Leiothrichidae, Monarchidae, Muscicapidae, Paradisaeidae, Paradoxornithidae, and Pellorneidae). According to modern concepts, this genus, along with seven more genera belongs to the “*Phroterothrix* generic group” incorporating archaic pterodectines with setae *ps3* anterior to adanal suckers in males (Mironov and Proctor 2009, Hernandes and Valim 2014). Twenty six out of the 28 species were arranged in three species groups: *megacaula* (3 species), *schizothyra* (4 species) and *wolffi* (19 species) (Gaud 1952, 1962, 1968, 1979, Mironov et al. 2008, 2010, 2012, Mironov and Proctor 2009, Mironov and Tolstenkov 2013, Constantinescu et al. 2014). The new species of *Proterothrix* described herein belongs to the *wolffi* species group, in having almost closed coxal fields III in males and parallel-sided terminal cleft in females.

## Materials and methods

The material used in the present paper was collected in Shnongrim (Meghalaya, India) in January 2014. The birds were captured using mist–nets, identified and visually checked for the presence and collection of mites and released back to the wild. Mite specimens were collected manually with a needle and placed in tubes with 96% ethanol. Later, in laboratory conditions, mite specimens were cleared in 90% lactic acid for 24 hours, and mounted on microscope slides in Hoyer’s medium. Drawings were made using an Olympus CX21 microscope, with a camera lucida drawing device. The bird specimens were identified according to Rasmussen and Anderton (2012) and Grimmett et al. (2011), and the taxonomy of birds follows Clements et al. (2016). The body setation of mites follows that of Griffiths et al. (1990) with the modifications by Norton (1998) concerning coxal setae, while the setation of legs follows Gaud and Atyeo (1996). The description of the new species of *Proterothrix* is given according to the current format used for species of pterodectine mites (Mironov and Fain 2003, Mironov 2006, Valim and Hernandes 2006, Mironov et al. 2008) and the measuring techniques of particular structures used were described by Mironov and Proctor (2009).

The full set of measurements is given for a holotype (male) and a range of measurements for corresponding paratypes. All measurements are in micrometers (μm). The holotype and paratypes of the new species are deposited in the Acarological Collection of the “Grigore Antipa” National Museum of Natural History, Bucharest, Romania (inventory numbers are given in brackets for all type specimens).

Three paratype specimens of *Proterothrix chachulae* sp. n. (two males ANA747, ANA748 and one female ANA749) were used to isolate DNA using DNAeasy Tissue Kit (Qiagen). All three specimens used for molecular analyses were mounted and kept as reference vouchers for morphological examination. The specimens preserved in ethanol 96% were transferred in 180μl ATL Buffer with 20 μl of Proteinase K and incubated overnight at 56ºC on a shaking thermoblock. After 24h, 5μl of Proteinase K were added and incubation was continued until 72h. For the rest of the protocol we followed the manufacturer specifications and the modification suggested by Dabert et al. (2008).

The 663bp fragment near the 5` terminus of the COI gene was used as DNA barcode region, amplified by PCR with the degenerate primers bcdF05 (5`- TTTTCTACHAAYCATAAAGATATTGC-3`) and bcdR04 (5`- TATAAACYTCDGGATGNCCAAAAAA-3`), according to Dabert et al. (2008). The PCRgenotyping reaction was performed in a 50 μL total volume containing DNA template, 1X Green GoTaq® Flexi Buffer, 2.5 mM MgCl2, each dNTP at 0.1 mM, each primer at 0.5 μM (the primers were M13 tailed) and 1.5 units of GoTaq® DNA polymerase (5U/μl) (Promega, Madison, USA). The PCR products were isolated from samples containing visible bands and sent for sequencing to Macrogen (Seoul, Korea).

Sequence chromatograms were edited and assembled with CodonCode Aligner version 3.7.1. Pairwise distances between sequences were computed with MEGA version 6 (Tamura et al. 2013) using K2P distance model (Kimura 1980). DnaSP v5 was used to obtain data about the genetic polymorphism in the studied specimens (Librado and Rozas 2009).

## Results

### Family Proctophyllodidae Trouessart et Mégnin, 1884

#### Subfamily Pterodectinae Park et Atyeo, 1971 Genus *Proterothrix* Gaud, 1968

##### Proterothrix chachulae

Taxon classificationAnimaliaPasseriformesProctophyllodidae

Constantinescu sp. n.

http://zoobank.org/9CC8B15E-BCFB-4288-9EAA-265ED462C931

**Figure 1.**
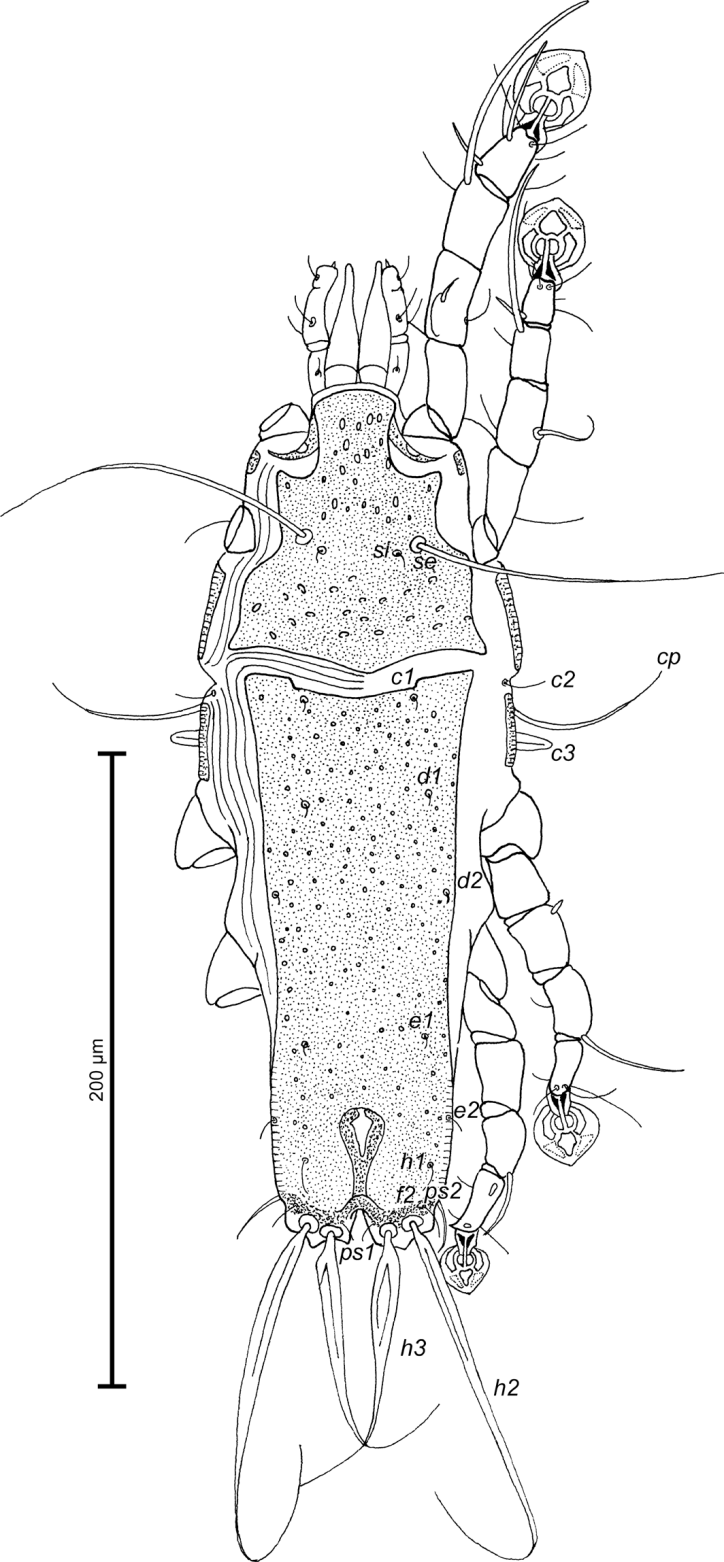
*Proterothrix chachulae* sp. n., male holotype: dorsal view of idiosoma.

**Figure 2.**
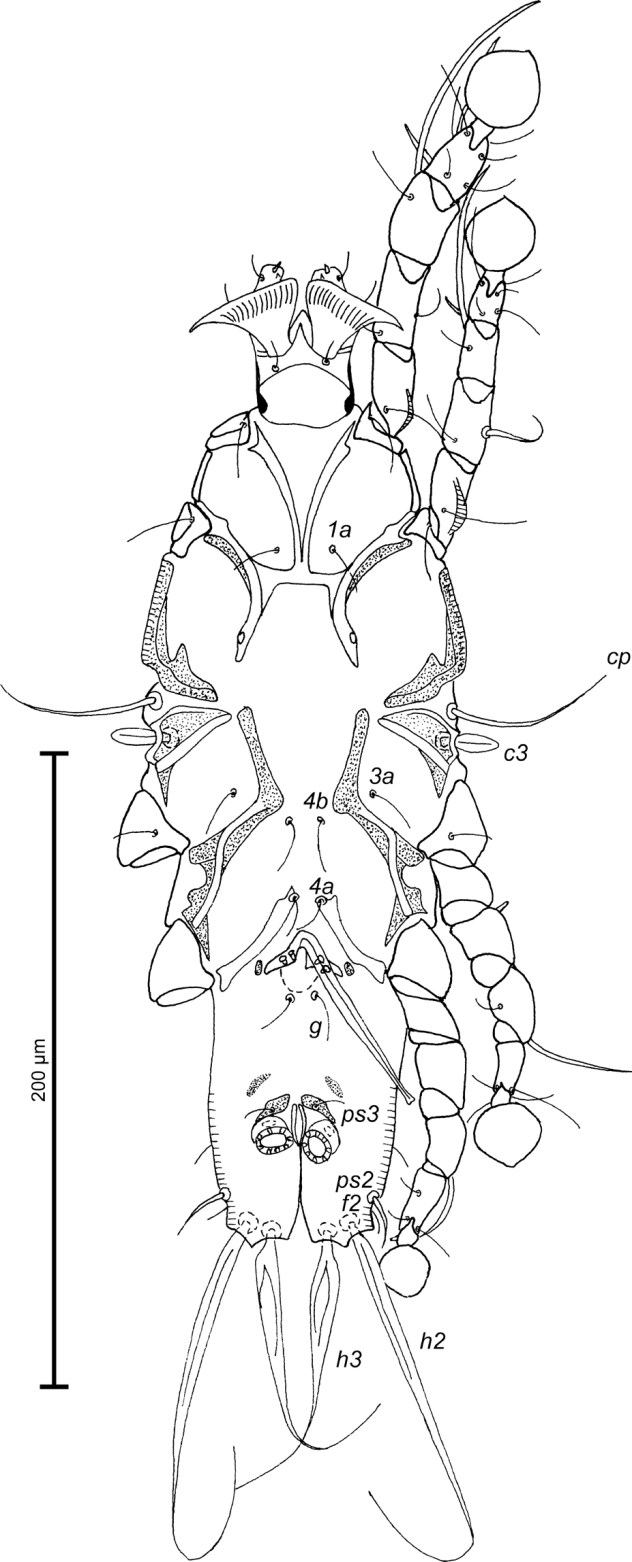
*Proterothrix chachulae* sp. n., male holotype: ventral view of idiosoma.

**Figure 3.**
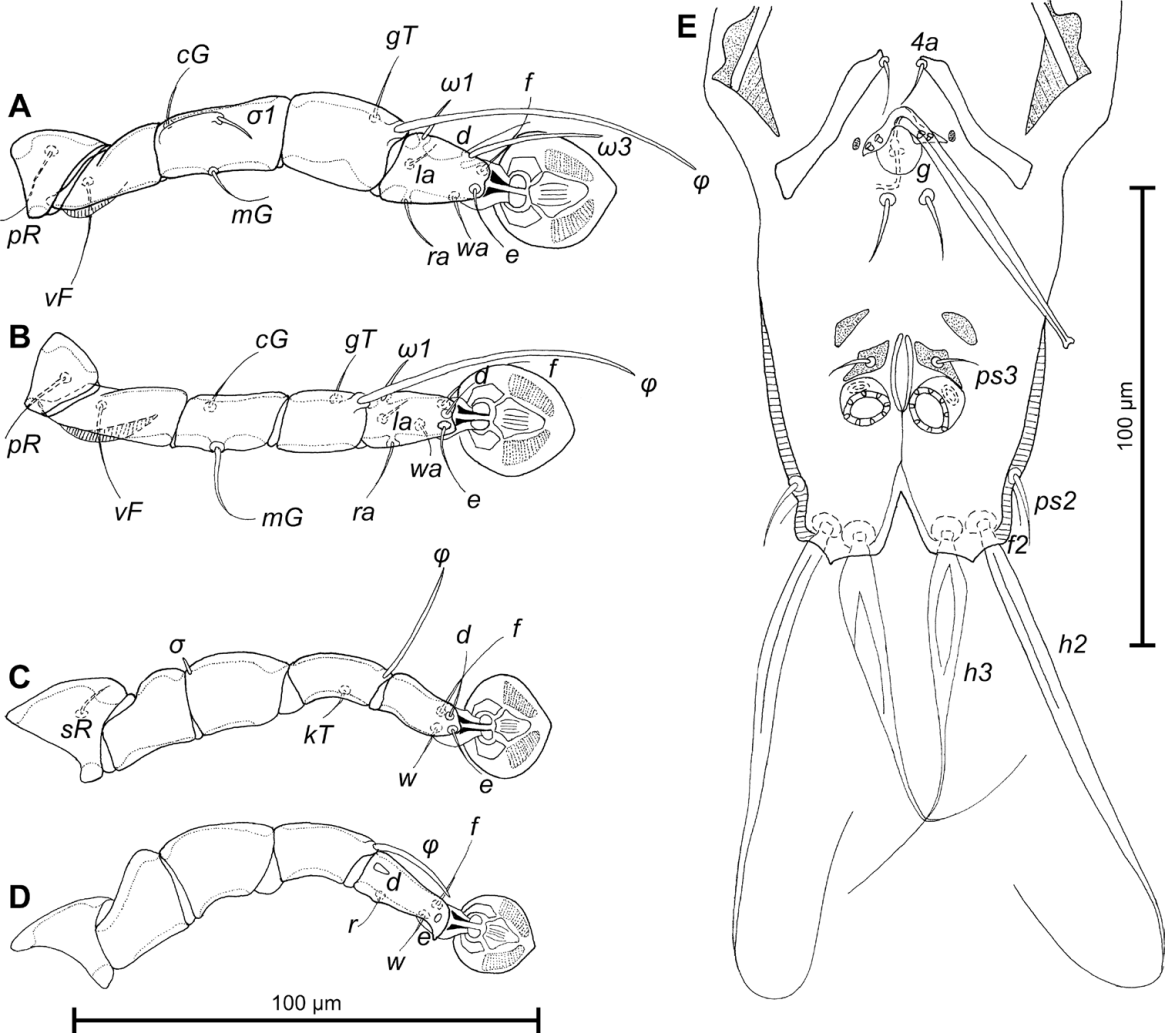
*Proterothrix chachulae* sp. n., **A–D** details of male legs, dorsal view: **A** leg I **B** leg II **C** leg III **D** leg IV **E** opisthosoma of male, ventral view.

**Figure 4.**
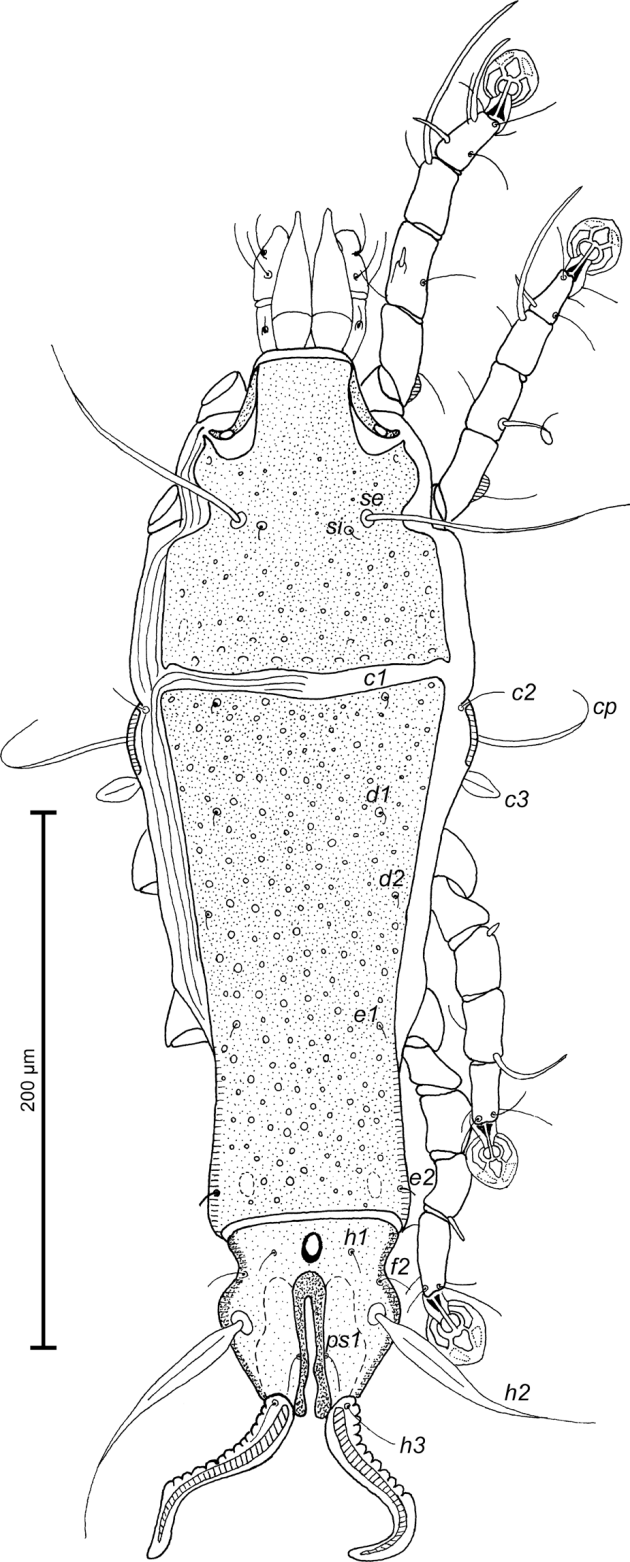
*Proterothrix chachulae* sp. n., female paratype: dorsal view of idiosoma.

**Figure 5.**
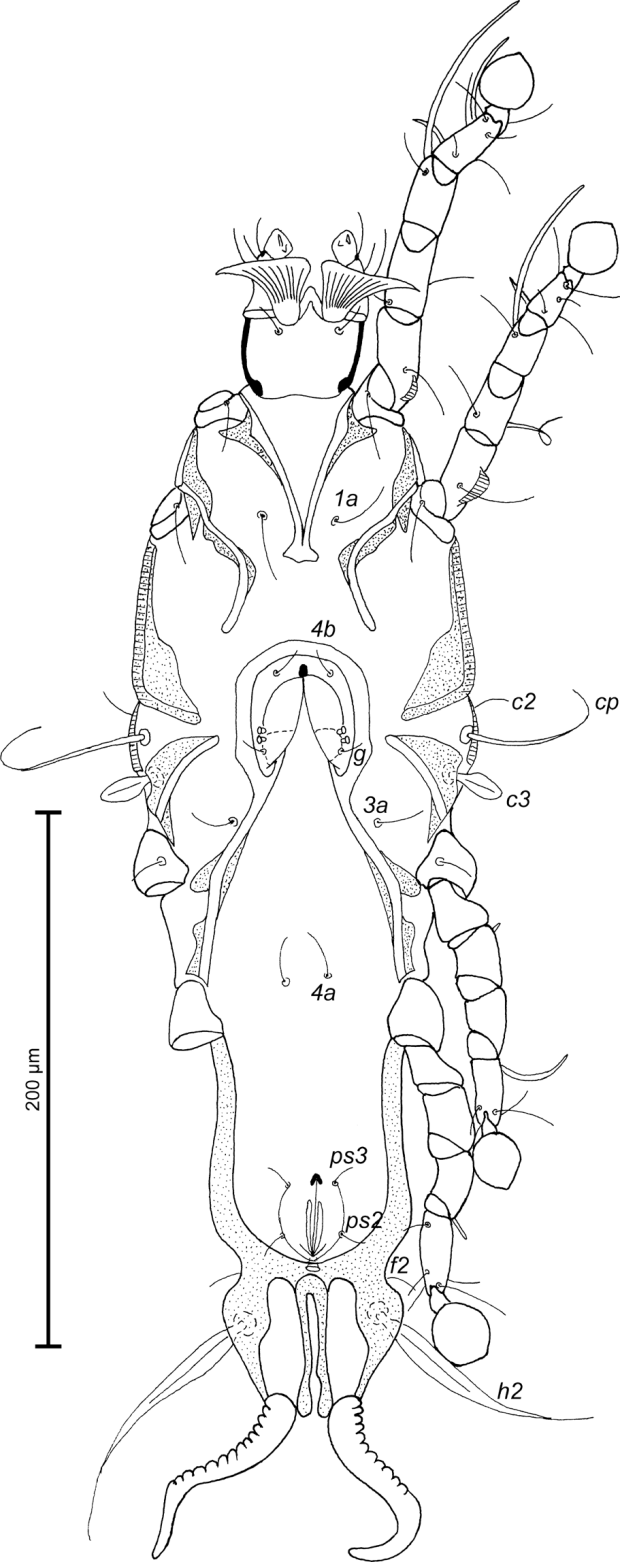
*Proterothrix chachulae* sp. n., female paratype: ventral view of idiosoma.

**Figure 6.**
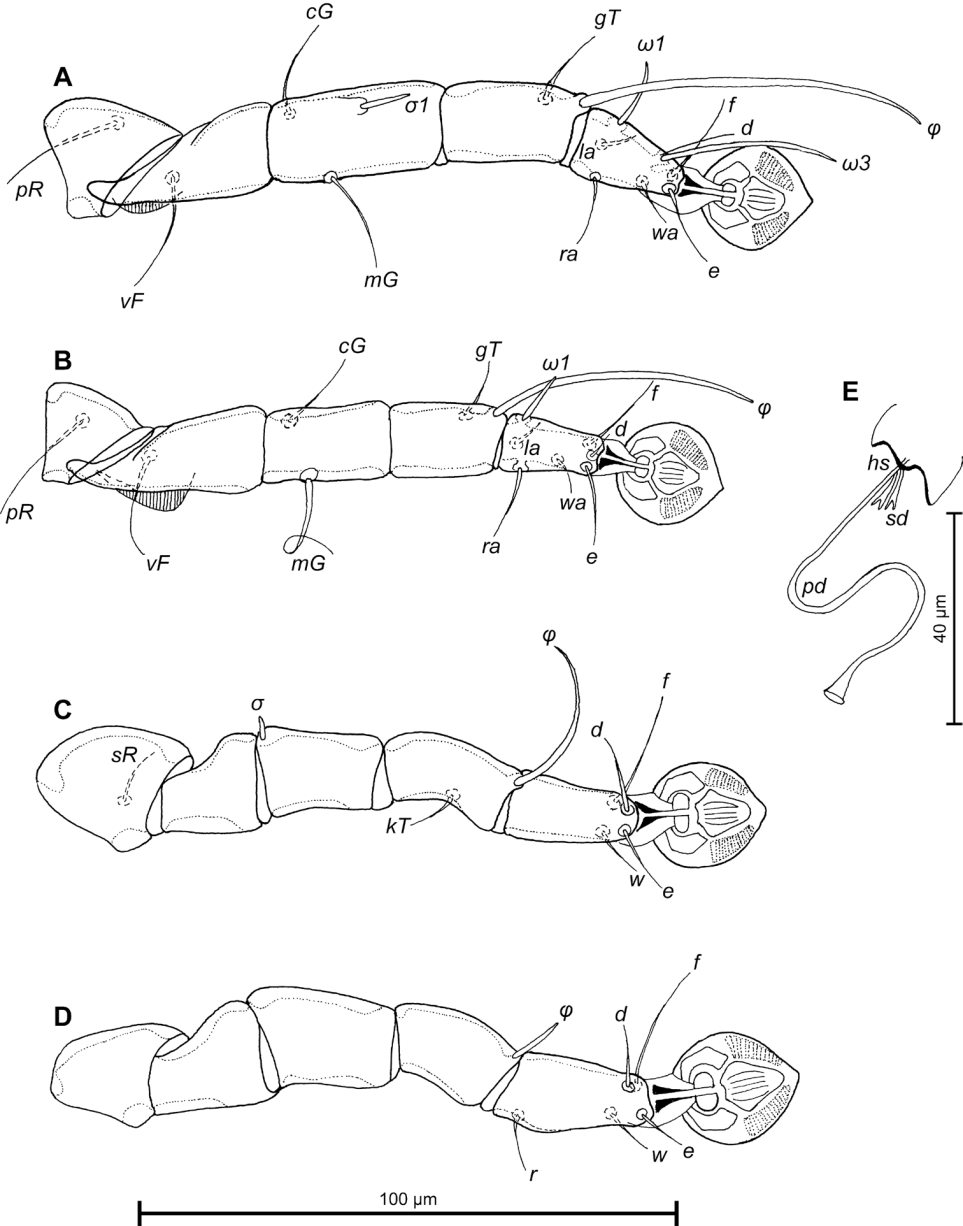
*Proterothrix chachulae* sp. n., **A–D** details of female legs, dorsal view: **A** leg I **B** leg II **C** leg III **D** leg IV **E** spermatheca and spermaducts of female. Abbreviations: hs–head of spermatheca; pd–primary spermaduct; sd–secondary spermaduct.

Figs 1 , 2 , 3 , 4 , 5 , 6

###### Type material.

Male holotype (ANA672), 6 male (ANA671, ANA673, ANA674, ANA675, ANA747(P2♂), ANA748(P1♂)) and 6 female (ANA676, ANA677, ANA678, ANA679, ANA680, ANA749(P1♀) paratypes, 27.01.2014, from the Large Niltava *Niltava grandis grandis* (Blyth) (Passeriformes, Muscicapidae); INDIA: Meghalaya, Jaintia Hills, Shnongrim village, (25°21'12.36"N, 92°31'3.06"E); 1151 m; subtropical forest; collector D. K. B. Mukhim.

###### Description.

MALE (Figs 1; 2; 3; holotype, range for 6 paratypes in parentheses): Pseudorutellar lobes with long and acute lateral extensions. Length of idiosoma 284 (284–288), width 104 (104–109), length of hysterosoma 184 (190–192). Prodorsal shield entire, anterolateral extensions short and with acute tips, lateral margins without incisions, posterior margin with wide blunt-angular extension, posterior angles well expressed, length 96 (94–96), width 82 (82–88), surface with ovate lacunae (Fig. 1). Scapular setae *se* separated by 36 (35–42). Scapular shields narrow. Humeral shields narrow, separated from outer sclerotized areas of epimerites III. Setae *cp* situated ventrally, setae *c2* filiform, situated dorsally, both pairs on striated tegument. Subhumeral setae *c3* lanceolate, 18 (18–20) × 6 (6–8). Hysteronotal shield with anterior margin concave, anterior angles rounded, distance from anterior margin to bases of setae *h3* 174 (180–186), greatest width in anterior part 74 (72–80), surface with small circular lacunae. Opisthosomal lobes roughly trapezoidal, short, each with angular median expansion on posterior margin, setae *h3* situated slightly posterior to setae *h2*. Terminal cleft V–shaped, 16 (16–20) in length; margins of terminal cleft without membranes. Supranal concavity clearly outlined, triangular. Setae *f2* slightly posterior to bases of setae *ps2*. Setae *h1* near lateral margins of opisthosoma. Setae *ps1* filiform, length 6 (5–8), situated near lateral margins of opisthosomal lobes, anterior to bases of setae *h3*. Setae *c1* present, setae *h3* flattened and enlarged in basal part, shorter than *h2*. Dorsal measurements: *c2*–*d2* 68 (64–68), *d2*–*e2* 70 (72–78), *e2*–*h3* 42 (40–46), *d1*–*d2* 33 (31–34), *e1*–*e2* 24 (20–26), *h1*–*ps2* 10 (7–11), *h2*–*h2* 36 (34–36), *h3*–*h3* 20 (20–22), *ps2*–*ps2* 44 (46–48).

Epimerites I fused into a V, posterior end connected with epimerites II by transverse sclerotized bands. Epimerites II long, with posterior ends free. Coxal field I closed, coxal field II open, coxal fields III almost closed, coxal fields IV with narrow sclerotized areas at bases of trochanters IV. Epimerites IVa present, well developed, their anterior tips bearing bases of setae *4a* (Fig. 2). Genital arch 12 (8–10) long, 18 (14–18) wide, basal sclerite of genital apparatus rounded posteriorly; aedeagus with bilobate tip 64 (64–69) long, extending to level of setae *ps3*. Genital papillae situated at level of genital arch. A pair of small ovoid sclerites located near tips of genital arch. Two pairs of adanal shields (median and anterolateral) represented by small triangular plates, setae *ps3* situated on median pairs. Anal suckers 12 (11–12) in diameter, corolla indented, with 8 small teeth. Ventral measurements: *3a*–*4b* 20 (18–22), *4b*–*4a* 30 (28–30), *4a*–*g* 30 (27–32), *g*–*ps3* 36 (37–40), *ps3*–*ps3* 17 (16–18), *ps3*–*h3* 36 (36–40).

Legs I longer than legs II, femora I and II with ventral crest (Fig. 3A, B). Seta *e* of tarsus I filiform. Setae *mG*II thickened basally, with filiform apex, setae *d* of tarsi II, III much shorter than corresponding setae *f.* Tarsus IV 23 (22–24) long, with apical claw-like process, setae *d, e* button-like, seta *d* bigger in diameter than *e*, situated in basal and apical parts of segment, respectively (Fig. 3D). Length of solenidia: *ω1*I 10 (10–14), *ω1*II 9 (8–10), *φ*I 56 (54–60), *φ*II 46 (42–44), *φ*III 25 (22–26), *φ*IV 22 (18–20).

FEMALE (Figs 4; 5; 6A–E; range for 6 paratypes): Pseudorutellar lobes with long and acute lateral extensions as in males. Length of idiosoma 392–396, width 125–132, and length of hysterosoma 270–280. Prodorsal shield entire, anterolateral extensions with rounded tips, lateral margins without deep incisions, posterior margin almost straight, length 117–121, width 107–110, surface with small circular lacunae in anterior part and bigger ovate and circular lacunae in posterior part (Fig. 4). Scapular setae *se* separated by 43–46. Scapular shields narrow. Humeral shields narrow, separated from epimerites III. Setae *c2* filiform, situated dorsally on striated tegument. Subhumeral setae *c3* lanceolate, 18–22 × 6–8. Anterior hysteronotal shield roughly rectangular, anterior and posterior margins concave, greatest length 189–190, greatest width in anterior part 99–101, surface with sparsely disposed circular lacunae. Length of lobar region 74–79, width at level of setae *h2* 63– 65. Terminal cleft parallel-sided, narrow, with almost touching margins posterior to level of setae *ps1*, length 45–52. Supranal concavity well developed, ovoid. Setae *h1* on lobar shield, at midlevel of supranal concavity; surface of lobar shield without ornamentation. Setae *h2* spindle-shaped, with terminal filament, 73–81 × 6–8. Setae *ps1* closer to inner margin of opisthosomal lobes, setae *h3* 17–18 long, about 1/3 from the length of terminal appendages. Dorsal measurements: *c2*–*d2* 83–84, *d2*–*e2* 99–105, *e2*–*h2* 44–47, *h2*–*h3* 32–35, *d1*–*d2* 34–47, *e1*–*e2* 40–42, *h1*–*h2* 28–32, *h2*–*ps1* 26–30, *h1*–*h1* 29–31, *h2*–*h2* 49–51.

Epimerites I fused as a V, with short lateral extensions. Coxal fields I, II with small sclerotized areas, epimerites IVa absent (Fig. 5). Translobar apodemes of opisthosomal lobes present, fused to each other anterior to terminal cleft. Epigynum horseshoe-shaped, greatest width 49–53. Secondary spermaducts short, about 10 long (Fig. 6E). Distance between pseudanal setae: *ps2*–*ps2* 20–23, *ps3*–*ps3* 16–18, *ps2*–*ps3* 16–20.

Legs I slightly longer than legs II; femora I, II with wide ventral crest; setae *mG*II thickened basally, with filiform apex. Length of solenidia: *ω1*I 9–11, *ω1*II 6–9, *φ*I 59–67, *φ*II 46–48, *φ*III 30–35, *φ*IV 5–7 (Fig. 6A–D).

###### Etymology.

This species is named in honor of Oana Mirela Chachula (a biologist, the National Museum of Romanian History, Romania), for her support of our research of ectoparasites of birds from Meghalaya (India).

###### Remarks.

*Proterothrix chachulae* sp. n. belongs to the *wolffi* species group by having almost closed coxal fields III in males and parallel-sided terminal cleft in females. Males of the new species differ from all known males of the genus by having the aedeagus with bilobate tip. Among all species of the genus, *Proterothrix chachulae* is closely related to *Proterothrix cyornis* Mironov and Tolstenkov, 2013 from *Cyornis tickelliae* Blyth (Passeriformes: Muscicapidae) by having the pseudorutelar lobes with acute lateral extensions in both sexes. Males of both species have the opisthosomal lobes roughly trapezoidal and short, setae *h3* flattened and enlarged in basal part, a similar general proportions of aedeagus and epimerites IVa well developed, their anterior tips bearing bases of setae *4a*. *Proterothrix chachulae* differs from that species by the following features: in males, the prodorsal shield has posterior margin with wide blunt-angular extension, the opisthosomal lobes have angular median expansion on posterior margin, a pair of small ovoid shields is located at the tips of the genital arch, seta *d* is bigger in diameter then *e* on tarsus IV; in females, the prodorsal shield bears in anterior part a few small circular lacunae, the lobar shield is without ornamentation, and the setae *h3* have about 1/3 from the length of terminal appendages. In males of *Proterothrix cyornis*, the prodorsal shield has posterior margin slightly convex, the opisthosomal lobes have posterior margin with a small median invagination, small sclerites near the tips of genital arch are absent, seta *d* and *e* on tarsus IV are subequal in diameter; in females, the prodorsal shield bears big ovate lacunae in anterior part, the lobar shield bears few circular lacunae in anterior part, and the setae *h3* have about 1/5 from the length of terminal appendages.

###### DNA barcode.

*Representative DNA sequences*. GenBank accession numbers for molecular voucher: ANA747 P2 male KY594726; ANA748 P1 male KY594724; ANA749 P1 female KY594725.

We sequenced a 660-pb fragment of the mitochondrial cytochrome c oxidase subunit I (COI) gene for two male and one female paratypes. In the resulted alignment we identified 8 variable sites. Two haplotypes were identified: H1 (ANA_748_P1_M and ANA_749_P1_F) and H2 (ANA_747_P2_M).

Intraspecific genetic distance between the analyzed specimens using K2P model is 0.8% (SE 0.3). All observed nucleotide substitutions were synonymous and did not change the amino acid sequence.

This reported genetic distance in the nucleotide sequence of the DNA barcode is comparable with genetic distances found for other Analgoidea species like *Proctophyllodes cetti* (0.87%) (Badek et al. 2008).

## Supplementary Material

XML Treatment for Proterothrix chachulae

## References

[B1] Atyeo WT (1973) Feather mites. Pp. 54–78. In: McClure HE and Ratanaworabhan N (Eds), Some ectoparasites of the birds of Asia. Jintana Printing Ltd. , Bangkok, Thailand, 219 pp.

[B2] Atyeo WT Braasch L (1966) The feather mite genus *Proctophyllodes* (Sarcoptiformes: Proctophyllodidae). Bulletin of the University of Nebraska State Museum 5: 1–354.

[B3] Badek A Dabert M Mironov SV Dabert J (2008) A new species of the genus *Proctophyllodes* (Analgoidea: Proctophyllodidae) from Cetti’s Warbler *Cettia cetti* (Passeriformes: Sylviidae) with DNA barcode data. Annales Zoologici 58(2): 397–402. https://doi.org/10.3161/000345408X326735

[B4] Clements JF Schulenberg TS Iliff MJ Roberson D Fredericks TA Sullivan BL Wood CL (2016) The eBird/Clements checklist of birds of the world [Internet]. Version 2016. http://www.birds.cornell.edu/clementschecklist/download/

[B5] Constantinescu IC Chişamera G Mukhim DKB Adam C (2014) Three new species of feather mite of the genus *Proterothrix* Gaud, 1968 (Analgoidea: Proctophyllodidae: Pterodectinae) from passerines in Meghalaya, North East India. Systematic Parasitology 89: 45–58. https://doi.org/10.1007/s11230-014-9508-1 25079815 10.1007/s11230-014-9508-1

[B6] Dabert J Ehrnsberger R Dabert M (2008) *Glaucalges tytonis* sp. n. (Analgoidea, Xolalgidae) from the barn owl *Tyto alba* (Strigiformes, Tytonidae): compiling morphology with DNA barcode data for taxon descriptions in mites (Acari). Zootaxa 1719: 41–52.

[B7] Gaud J (1952) Sarcoptides plumicoles des oiseaux de Madagascar. Mémoires de L'Institut scientifique de Madagascar 7(1): 81–107.

[B8] Gaud J (1962) Sarcoptiformes plumicoles (Analgesoidea) parasites d'oiseaux de I'Ile Rennell. The Natural History of Rennell Island, British Solomon Islands 4: 31–51.

[B9] Gaud J (1968) Sarcoptiformes plumicoles (Analgoidea) parasites d'oiseaux de I'Ile Rennell. The Natural History of Rennell Island, British Solomon Islands 5: 121–151.

[B10] Gaud J (1979) Sarcoptiformes plumicoles des oiseaux Coraciiformes d'Afrique. II. Parasites des Alcedinidae. Revue de Zoologie Africaines 93: 245–266.

[B11] Gaud J Atyeo WT (1996) Feather mites of the world (Acarina, Astigmata): the supraspecific taxa. Annalen Zoologische Wetenschappen, Musée Royal de L'Afrique Centrale, Annales Sciences Zoologiques 277: 1–191.

[B12] Griffiths DA Atyeo WT Norton RA Lynch CA (1990) The idiosomal chaetotaxy of astigmatid mites. Journal of Zoology 220: 1–32. https://doi.org/10.1111/j.1469-7998.1990.tb04291.x

[B13] Grimmett R Inskipp C Inskipp T (2011) Helm Field Guides: Birds of the Indian Subcontinent. Christopher Helm, London, 528 pp.

[B14] Hernandes FA Valim MP (2014) On the identity of two species of Proctophyllodidae (Acari: Astigmata: Analgoidea) described by Herbert F. Berla in Brazil, with a description of *Lamellodectes* gen. nov. and a new species. Zootaxa 3794(1): 179–200. https://doi.org/10.11646/zootaxa.3794.1.8 24870318 10.11646/zootaxa.3794.1.8

[B15] Kimura M (1980) A simple method for estimating evolutionary rate of base substitutions through comparative studies of nucleotide sequences. Journal of Molecular Evolution 16: 111–120. https://doi.org/10.1007/BF01731581 7463489 10.1007/BF01731581

[B16] Librado P Rozas J (2009) DnaSP v5 - A software for a comprehensive analysis of DNA polymorphism data. Bioinformatics 25: 1451–1452. https://doi.org/10.1093/bioinformatics/btp187 19346325 10.1093/bioinformatics/btp187

[B17] Mironov SV (2006) Feather mites of the genus *Montesauria* Oudemans (Astigmata: Proctophyllodidae) associated with starlings (Passeriformes: Sturnidae) in the Indo- Malayan region, with notes on systematic of the genus. Acarina 14(1): 21–40.

[B18] Mironov SV Diao W Zhang Y Zhang C Yan Z (2008) A new mite species of the genus *Proterothrix* Gaud (Astigmata, Proctophyllodidae) from *Ficedula zanthopygia* (Hay) (Passeriformes: Muscicapidae) in China. Acarina 16: 31–38.

[B19] Mironov SV Fain A (2003) New species of feather mite subfamily Pterodectinae (Astigmata: Proctophyllodidae) from African passerines (Aves: Passeriformes). Bulletin et Annales de la Societe Royale Belge d'Entomologie 139: 75–91.

[B20] Mironov SV Proctor HC (2009) Feather mites of the genus *Proterothrix* Gaud (Astigmata: Proctophyllodidae) from parrotbills (Passeriformes: Paradoxornithidae) in China. Journal of Parasitology 95: 1093–1107. http://dx.doi.org/10.1645/GE-1961.1 19281277 10.1645/GE-1961.1

[B21] Mironov SV Tolstenkov OO (2013) Three new feather mites of the subfamily Pterodectinae (Acari: Proctophyllodidae) from passerines (Aves: Passeriformes) in Vietnam. Proceedings of Zoological Institute of Russian Academy of Sciences 317(1): 11–29.

[B22] Mironov SV Literak I Čapek M (2008) New feather mites of the subfamily Pterodectinae (Acari: Astigmata: Proctophyllodidae) from passerines (Aves: Passeriformes) in Mato Grosso do Sul, Brazil. Zootaxa 1947: 1–38.

[B23] Mironov SV Literak I Čapek M Koubek P (2010) New species of the feather mite subfamily Pterodectinae (Astigmata, Proctophyllodidae) from passerines in Senegal. Acta Parasitologica 55(4): 399–413. https://doi.org/10.2478/s11686-010-0051-1

[B24] Mironov SV Literak I Hung MN Čapek M (2012) New feather mites of the subfamily Pterodectinae (Acari: Proctophyllodidae) from passerines and woodpeckers (Aves: Passeriformes and Piciformes) in Vietnam. Zootaxa 3440: 1–49.

[B25] Norton AR (1998) Morphological evidence for the evolutionary origin of Astigmata (Acari: Acariformes). Experimental and Applied Acarology 22: 559–594. https://doi.org/10.1023/A:1006135509248

[B26] Orwig KR (1968) The genera and species of the feather mite subfamily Trouessartiinae except *Trouessartia* (Acarina: Proctophyllodidae). Bulletin of the University of Nebraska State Museum 8: 1–187.

[B27] Park CK Atyeo WT (1971) A generic revision of the Pterodectinae, a new subfamily of feather mites (Sarcoptiformes: Analgoidea). Bulletin of the University of Nebraska State Museum 9(3): 40–88.

[B28] Rasmussen PC Anderton JC (2012) Birds of South Asia. The Ripley Guide. Volumes 1 and 2. Second Edition. National Museum of Natural History – Smithsonian Institution, Michigan State University and Lynx Edicions, Washington, D.C., Michigan and Barcelona, vol. 1: 684 pp., vol. 2: 378 pp.

[B29] Tamura K Stecher G Peterson D Filipski A Kumar S (2013) MEGA6: Molecular Evolutionary Genetics Analysis version 6.0. Molecular Biology and Evolution 30: 2725–2729. https://doi.org/10.1093/molbev/mst197 24132122 10.1093/molbev/mst197 PMC3840312

[B30] Valim MP Hernandes FA (2006) Redescription of four species of the feather mite genus *Pterodectes* Robin, 1877 (Acari: Proctophyllodidae: Pterodectinae) described by Herbert F. Berla. Acarina 14: 41–55.

